# Dopamine D2S/D2L Receptor Regulation of Alcohol‐Induced Reward and Signalling

**DOI:** 10.1111/adb.70093

**Published:** 2025-11-15

**Authors:** Mohd Tayyab, Toshikuni Sasaoka, Manabu Abe, Nae Saito, Kenji Sakimura, Anetta Ninan, YanYan Wang

**Affiliations:** ^1^ Department of Cellular and Molecular Biology The University of Texas at Tyler School of Medicine Tyler Texas USA; ^2^ Department of Pharmaceutical Sciences and Health Outcome The University of Texas at Tyler Fisch College of Pharmacy Tyler Texas USA; ^3^ Department of Comparative and Experimental Medicine, Brain Research Institute Niigata University Niigata Japan; ^4^ Department of Animal Model Development, Brain Research Institute Niigata University Niigata Japan

**Keywords:** Akt, alcohol use disorder, cannabinoid receptors, dopamine D2L receptor, dopamine D2S knockout mice, dopamine D2S receptor

## Abstract

Dopamine D2 receptor (D2R)‐mediated signalling is involved in reward, motivation and alcohol use disorder. Perturbation of the D2R system may influence an individual's response to alcohol. Alternative splicing of the D2R gene generates two isoforms: D2 long form (D2L) and D2 short form (D2S). It is unclear whether differences in the expression of D2L and D2S influence alcohol's effects. Here we examined if altered expression levels of either D2R isoform would influence alcohol effects on reward‐related behaviour and relevant signalling pathways using knockout (KO) mice expressing either D2R isoform. We found that D2L KO mice (expressing only D2S) displayed a strong alcohol conditioned place preference (CPP) compared to WT mice and D2S KO mice (expressing only D2L). Alcohol exposure caused a downregulation of cannabinoid 1 receptors (CB1R) expression but an upregulation of cannabinoid 2 receptors (CB2R) expression in the striatum of D2L KO mice but not in WT and D2S KO mice. In addition, alcohol exposure resulted in decreased Akt phosphorylation selectively in D2L KO mice. Furthermore, the gene expressions of tyrosine hydroxylase (TH), Arc and RETN were also selectively downregulated in D2L KO mice following chronic alcohol exposure. Our results indicated that an alteration in the expression of D2S vs. D2L had significant impacts on alcohol‐induced reward and gene expression changes in the striatum. These findings suggest that the increased expression level of D2S to D2L may be a pathophysiological mechanism for developing alcoholism, possibly through triggering a cascade of changes in the cannabinoid‐Akt signalling pathway and relevant signalling network.

## Graphical abstract

D2L KO mice (expressing only D2S) displayed a strong alcohol conditioned place preference compared to WT and D2S KO mice (expressing only D2L). Alcohol exposure caused downregulation of cannabinoid 1 receptors expression and decreased Akt phosphorylation, but upregulation of cannabinoid 2 receptors expression in the striatum of D2L KO mice, but not in WT and D2S KO mice. Moreover, the gene expressions of tyrosine hydroxylase, Arc, and RETN were selectively downregulated in D2L KO mice following chronic alcohol exposure.

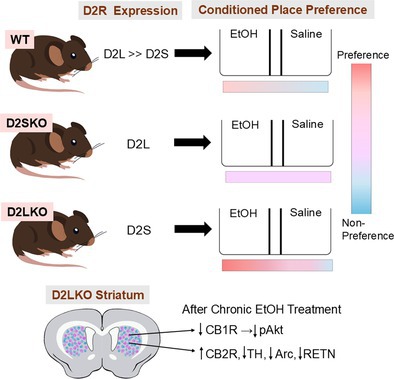

## 1 Introduction

Alcohol is a widely consumed psychoactive substance globally. In the United States, more than 85% of the population report lifetime use of alcohol [1, 2]. The WHO reports more than 3 million deaths every year worldwide due to harmful use of alcohol. Ample studies suggest that excessive alcohol consumption leads to alterations in the brain's neural circuits responsible for emotion, cognition and motor planning. Existing treatments are inadequate. The effort to understand factors contributing to excessive drinking behaviour and identify novel targets is crucial for developing new therapeutic approaches for intervening in alcohol abuse and dependence and reducing health care costs.

Compelling evidence has indicated that addictive substances, including alcohol, can stimulate the surge of dopamine (DA) in the brain reward system, including the striatum [3, 4]. DA exerts its effects by binding with subtypes of DA receptors. Studies have shown that the dopamine D2 receptor (D2R) plays a key role in the predisposition for excessive alcohol intake in humans and animal models, and perturbation of the D2R system may influence an individual's response to alcohol use, leading to addiction.

Imaging studies in human brains have demonstrated that chronic alcohol consumption alters striatal DA and D2R systems [5]. These studies have identified several alterations in the dopaminergic system in individuals with alcohol use disorder (AUD): increased DA release and/or decreased D2R availability in the striatum [6, 7, 8] or disruption of the striatal network [9]. In the extrastriatal areas, a recent study has found that D2R availabilities in the cingulate cortex are higher in people at high risk for developing alcoholism [10]. Microdialysis studies in rodents have reported increased DA release in both the ventral and dorsal striatum after alcohol exposure [11, 12]. A study using fast‐scan cyclic voltammetry has shown that alcohol alters DA release and D2R function in the striatum of primates (macaques) [13].

Research has shown that pharmacological agents with antagonistic activity at D2R can reduce alcohol consumption or alcohol‐induced behaviours. Microinjection of a D2R antagonist into the nucleus accumbens (NAc, the ventral striatum) decreases alcohol consumption in rats [14]. A clinical study demonstrates that low doses of the D2R antagonist haloperidol reduce the reinforcing effects of alcohol in social drinkers [15]. Another study shows that (‐)‐OSU6162 (OSU), a DA stabilizer acting as either a partial D2R agonist or D2R antagonist, can reduce alcohol consumption in rats [16] and alcohol preference in humans [17]. Moreover, aripiprazole, a partial D2R agonist, can attenuate alcohol‐induced conditioned place preference (CPP) in mice [18] and alcohol‐elicited striatal activation in humans [19].

The effects of D2R on alcohol mechanism of actions are likely mediated by selected signalling pathway genes. Accumulating evidence indicates that the D2R system interacts with the cannabinoid receptor system including cannabinoid type 1 receptor (CB1R) and cannabinoid type 2 receptor (CB2R) in the brain. Both CB1R and CB2R have been shown to be colocalized with D2R in neurons and astrocytes in the striatum [20, 21], providing an anatomical basis for the functional interactions between CB1R/CB2R and D2R. Several studies suggest that the interactions between the D2R and endocannabinoids receptor systems in the striatum play a role in alcoholism [22, 23]. Stimulation of D2R can modulate the effect of CB1R on intracellular cAMP production through G proteins [24, 25]. The serine/threonine protein kinase Akt pathway can be regulated by D2R as well as CB1R in mouse brain including the striatum [26, 27]. The activity‐regulated cytoskeleton‐associated protein (Arc) is a key protein involved in synaptic plasticity related to reward behaviour and drug addiction [28]. A study has shown that the reduction of Arc in mice results in reduced dopaminergic responses in the prefrontal cortex and increased DA/D2R‐mediated responses in the striatum [29].

Alternative splicing generates two D2R isoforms, namely, the D2 short form (D2S) and D2 long form (D2L) [30]. We previously generated DA D2L knockout (D2L KO) mice, which still express a functional D2S isoform at a level similar to the native level of total D2R in wild‐type (WT) mice [31]. D2L is generally the predominant isoform expressed in WT mice [31]. Over the years, we demonstrated that D2S and D2L play differential roles in regulating motor function, dopaminergic drug‐induced effects, neuronal excitability, and so on; for example, [32, 33, 34, 35]. In this study, we described the generation of D2S KO mice, which still express the D2L isoform at a level similar to the total D2R level in WT mice. The mice expressing either D2R isoform allow us to investigate the function and signalling of D2R in an isoform specific manner.

The functional relevance of the altered expression of the long or short isoform of D2R on alcohol‐induced effects remains largely unknown. The aims of this study were to determine whether changes in the expression levels of individual D2R isoforms affect alcohol's effects on reward‐related behaviour and relevant signalling pathways. We first explored the influence of individual D2R isoforms on alcohol reward effect as well as alcohol effect on motor coordination as a comparison. We then assessed if the alterations in D2S or D2L expression levels would have an impact on modulating the cannabinoid receptor system and selected genes of interest in mouse striatum following chronic alcohol exposure using genetically modified KO mice expressing either D2L (D2S KO) only or D2S (D2L KO) only. The cannabinoid receptor system and several genes were chosen because they have been shown to interact with the D2R signalling and/or be involved in AUD. This study would enrich our understanding of molecular and cellular mechanisms that are involved in the development of alcohol abuse and addiction.

## 2 Materials and Methods

### 2.1 Animals

D2L KO mice were generated and backcrossed to a C57BL/6 N background as previously described [31]. D2S KO mice were generated at Brain Research Institute, Niigata University, Japan, according to the method described by Hayashi et al. [36]. Briefly, to generate D2S KO mice, we designed genetic modifications so that D2L‐type mRNA containing exons 5, 6 and 7 is always expressed and D2S‐type mRNA lacking exon 6 is not expressed. Mutually exclusive pre‐mRNA splicing of adjacent exons is determined by the proximity of the splice donor site of the upstream exon to the branch point of the downstream exon. If the length of the proximity is less than 51 nucleotides, the branch point of the downstream exon is not used, and the branch point of the further downstream exon is used for splicing [36]. To create the targeting vector, we designed an artificial intron to meet this requirement.

**FIGURE 1:**
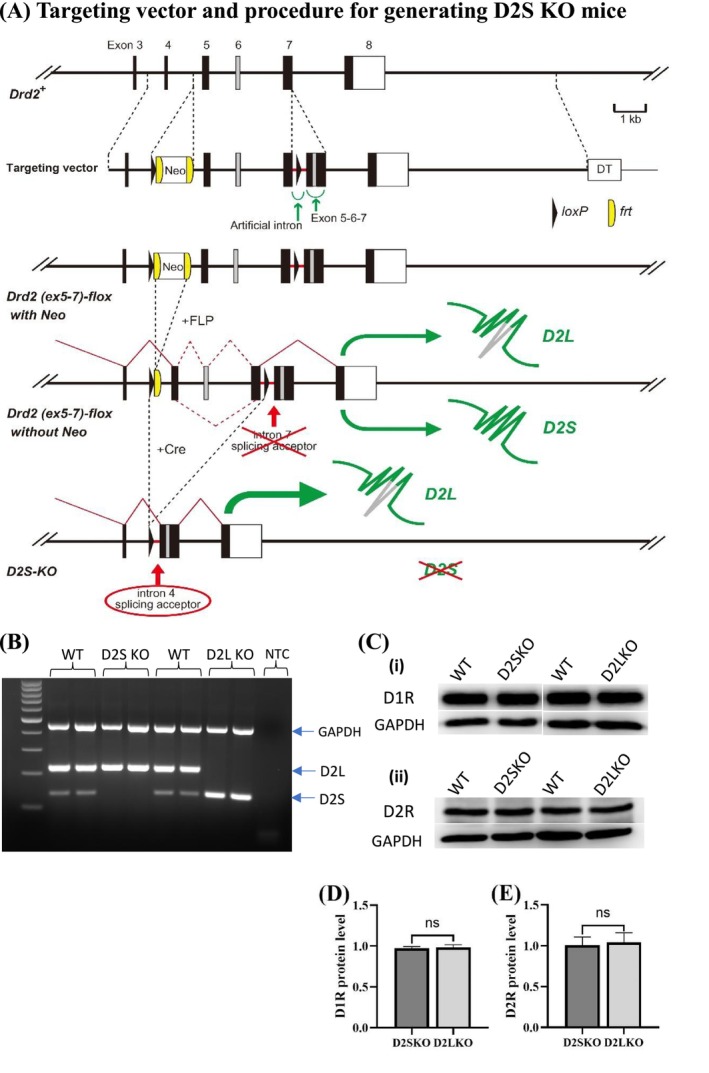
Generation of D2S KO mice. (A) The targeting vector and procedure for generating D2S KO mice. The DNA fragment containing loxP‐frt‐Neo gene‐frt was inserted into intron 4 of the Drd2 (i.e., D2R) gene and the DNA fragment containing the artificial intron and loxP‐exon 5, 6 and 7 sequence without introns 5 and 6 was inserted into the intron 7 of the Drd2 gene. This artificial intron was designed not to act as a splicing acceptor during splicing of exon 7. In this modification both D2L and D2S isoforms were designed to be expressed before Cre‐loxP recombination. After Cre‐loxP recombination, the regions between loxP in intron 4 and loxP in intron 7 was deleted, and the expressed mRNA always became the D2L isoform type, which contains exons 5, 6 and 7. The Drd2 (ex5–7)‐flox with Neo gene mice were generated by homologous recombination of ES cells using the targeting vector, generation of chimeric mice using ES cells and breeding of the chimeric mice. The Drd2 (ex5‐7)‐flox without Neo gene mice were generated by bred with FLP recombinase‐expressing mice to remove the frt‐Neo gene‐frt sequences. Next the Drd2 (ex5‐7)‐flox without Neo gene mice were bred with Cre recombinase‐expressing mice to generate D2S KO heterozygous mice. (B) Identifying the expression of D2L and D2S mRNAs in three genotypes of mice by RT‐PCR analysis. In WT mice (lanes 2–3 and 6–7), two D2R isoforms were detected with that the expression of D2L (size: 222 bp) is much higher than that of D2S (size: 135 bp). In D2S KO mice (lanes 4 and 5), only D2L mRNA (222 bp) was expressed. In D2L KO mice (lanes 8 and 9), only D2S mRNA (135 bp) was expressed. NTC (lane 10) is a no template control. GAPDH was used as a reference gene. The size marker (the first line of the gel picture) is a 100‐bp DNA ladder. (C) Assessing the expression levels of dopamine D1R and D2R proteins by Western blot analysis. (i) D1R expression in WT, D2S KO and D2L KO. (ii) D2R expression in WT, D2S KO and D2L KO mice. GAPDH was used as a loading control. mRNAs and proteins were isolated from the striatum of the mice. (D) Bar graph showing the quantification of Western blot for D1R expression shown in (C). D1R expression levels in KO mice are expressed as a ratio deviation from the average D1R expression level of WT mice. ns = not significant (*p* = 0.81; unpaired Student's *t*‐test). (E) Bar graph showing the quantification of Western blot for D2R expression shown in (C). D2R expression levels in KO mice are expressed as a ratio deviation from the average D2R expression level of WT mice. ns = not significant (*p* = 0.83).

The procedure for generating D2S KO mice is shown in Figure 1A and as follows. First, the artificial intron containing a loxP sequence linked to the combined exons 5‐6‐7 was inserted downstream of exon 7 of the mouse Drd2 (D2R) gene (Figure S1). This artificial intron was designed not to act as a splicing acceptor (a splice branch site) during splicing of exon 7. Therefore, before Cre‐loxP recombination, both D2L and D2S isoforms were expressed, but after Cre‐loxP recombination, the regions between loxP in intron 4 and loxP in intron 7 were deleted, and the expressed mRNA always became the D2L type, which contains exons 5, 6 and 7 following exon 4. The targeting vector was constructed by linking a 1.7 kb of 5′ homology arm, a 1.7 kb DNA fragment containing lox‐frt‐Neo gene‐frt, the region from intron 4 to exon 7 of the mouse Drd2 gene, the artificial intron, the combined exons 5‐6‐7 and a 9.3 kb of 3′ homology arm containing the region between intron 7 and the downstream region of exon 8 of the Drd2 gene. The sequence data of the region from exon 5 to the combined exons 5‐6‐7 of the targeting vector are shown in Figure S1. Second, for the generation of Drd2 (ex5–7)‐flox with Neo gene mice, this targeting vector was transfected into RENKA embryonic stem (ES) cells (C57BL/6 strain) by electroporation, and accurate homologous recombinant ES clones were obtained. The ES clones were microinjected into ICR mouse blastocysts to obtain chimeric mice. The chimeric mice were crossed with C57BL/6 mice to produce heterozygous mice of the Drd2 (ex5–7)‐flox with Neo gene. Third, by breeding with FLP recombinase‐expressing mice to remove the regions containing frt‐Neo gene‐frt by FLP‐frt recombination, heterozygous mice of Drd2 (ex5‐7)‐flox without Neo gene were obtained. After breeding heterozygous mice of the Drd2 (ex5–7)‐flox without Neo gene with Cre recombinase‐expressing mice, D2S KO heterozygous mice were generated. D2S KO heterozygous mice were bred to obtain D2S KO homozygous mice which are on a C57BL/6 N background.

WT, D2SKO and D2LKO male mice (8–10 weeks old) were used. Animal experiments were approved by the Institutional Animal Care and Use Committee (IACUC) of the University of Texas at Tyler Health Science Center and conducted following the US National Institute of Health Guide for the Care and Use of Laboratory Animals and by the President of Niigata University in accordance with the guidelines of the National Institutes of Health and the Ministry of Education, Culture, Sports, Science and Technology (MEXT) of Japan through the review of the IACUC of Niigata University.

### 2.2 Open‐Field Test (OFT)

Locomotor activity and rearing behaviour were assessed in a 17 in. × 17 in. open field chamber as previously described [37]. The chamber was equipped with a 16‐beam infrared array system (MED Associates Inc., St. Albans, VT). Mouse locomotion and rearing behaviour were monitored for 15 min. Data were collected by a computer and quantified as total distance travelled, total distance travelled in central zone, ambulatory counts and vertical counts.

### 2.3 Elevated Zero‐Maze Test

Anxiety‐related behaviour was assessed using the elevated zero‐maze test as previously described [38]. The elevated zero‐maze consisted of a dark grey PVC circular platform (60‐cm diameter, 7‐cm width) elevated 60 cm above the floor. The maze was divided into four equal quadrants, with two open quadrants and two enclosed by dark grey PVC walls (19 cm high). During testing, a mouse was placed in an open quadrant facing a closed quadrant and observed for 5 min. Data analysis was done by assessing the percentage of time spent in open areas.

### 2.4 Rotarod Test

Motor coordination was assessed using the rotarod test as previously described [38]. The test was conducted using a Rotarod apparatus (Ugo Basile, Stoelting). The accelerating rotarod treadmill's speed increased from 2 to 40 rpm over 5 min (the trial's cutoff time). Each mouse received two trials with a 2‐h interval, and their average time staying on the rod was used for data analysis. A timer automatically recorded the duration each mouse remained on the rotating rod. The same mice were used for locomotor activity and baseline rotarod experiments.

### 2.5 CPP

The automated place preference apparatus consists of two distinct chambers (10 × 13 × 13 cm, W × D x H) with unique visual and tactile cues that are interconnected by a central grey corridor. The sessions were monitored via weight sensors in the chambers that were connected to a computer using the software (PPCWin) from the manufacturer (Panlab, Harvard Bioscience, MA, USA). The CPP procedure was similar to that described previously [39]. Briefly, during the first 3 days of the preconditioning period, mice were placed in the central grey section with the doors opened and allowed to freely explore the entire apparatus for 20 min, and their baseline chamber preference was determined on day three. During the conditioning period, testing mice received saline injections intraperitoneally (i.p.) on Days 4, 6, 8 and 10 and were placed in their preferred compartment for 20 min with doors closed and received EtOH injection (2 g/kg, i.p.) on Days 5, 7, 9 and 11 and were placed in their nonpreferred compartment for 20 min with doors closed. Control mice received daily saline with alternating compartment placement. On Day 12 (postconditioning), all mice were reintroduced to the central corridor of the apparatus with doors open to allow them to freely explore the apparatus for 20 min. Time spent in each compartment was recorded. Changes in time spent in each compartment between postconditioning and preconditioning were calculated. The change in time that testing mice spent in the saline‐paired compartment is the total time (20 min) minus the time spent in the EtOH‐paired compartment.

### 2.6 Drug Treatment and Experimental Design

In the CPP experiment, male mice (8–10 weeks old) were randomly assigned to ethanol (EtOH) and control groups (Saline) in the following six groups: WT Saline, WT EtOH, D2SKO Saline, D2SKO EtOH, D2LKO Saline and D2LKO EtOH. EtOH solution (20% v/v) was made fresh weekly by diluting 200 proof EtOH (absolute) with sterile physiological saline (0.9% NaCl). The procedure for the CPP test was shown in the Experimental timeline diagram (A) below. After the CPP test, mice were treated with EtOH or saline for an additional 21 days (see Experimental timeline diagram (B)). Briefly, the EtOH groups of mice received daily injections of EtOH (2 g/kg, i.p.) for 21 consecutive days, while the control groups of mice received daily injections of an equal volume of saline. Injections were administered in their home cages in the morning. The injection sites were alternated across the midline each day to minimize skin irritation. EtOH dosage was adjusted daily based on the mice's weight. After 21 days of EtOH exposure, a rotarod test was conducted on day 21 in the afternoon, and the striatal tissues were dissected on day 22 to isolate mRNA or protein for qPCR or Western blot analyses. All mice used for locomotor and baseline rotarod experiments, plus additional mice that had not been treated with any drugs were used for CPP. After CPP, the mice received EtOH in CPP received EtOH injection for 3 weeks, and the mice received saline in CPP received saline injection for 3 weeks, and the behavioural test and molecular assessments were conducted on these mice.

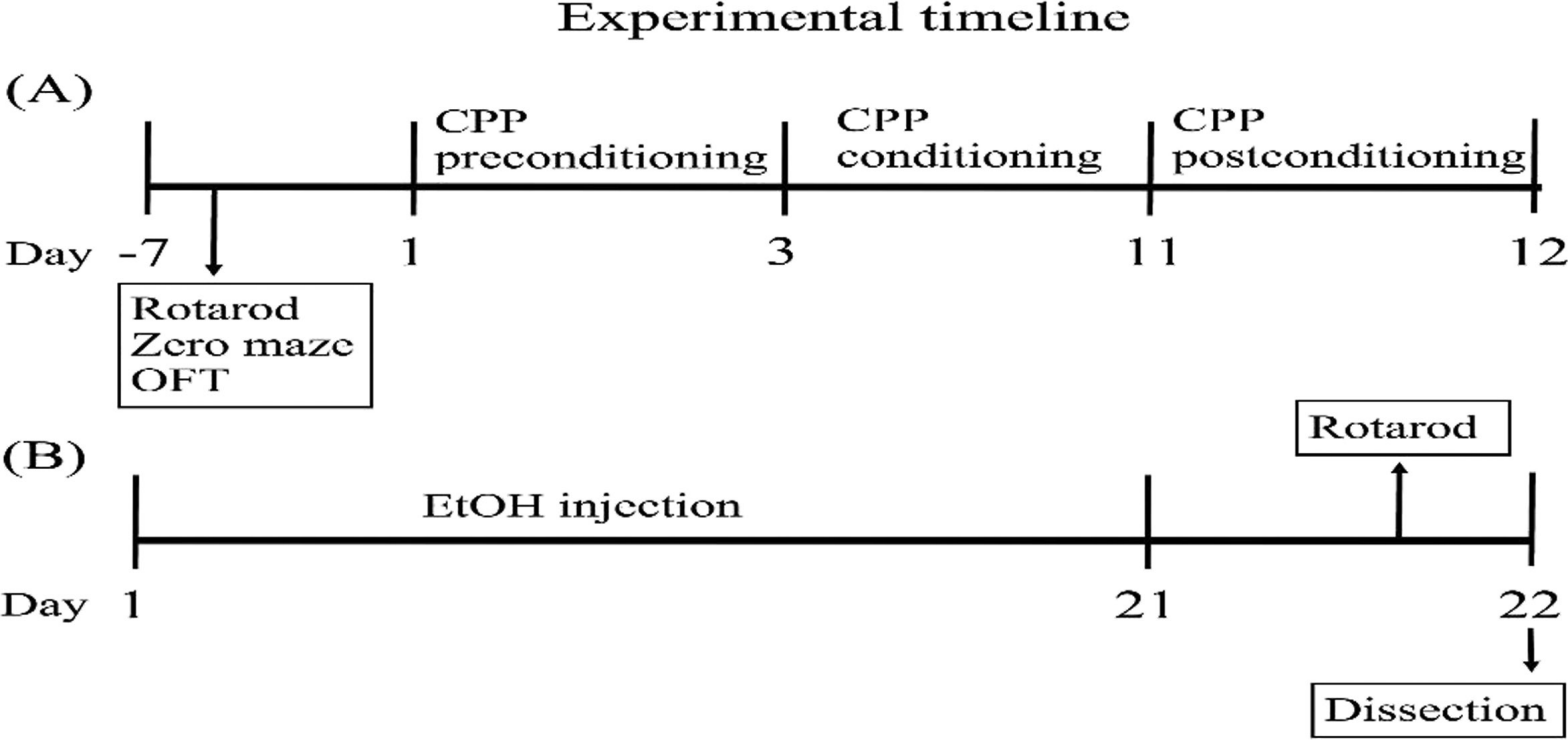

### 2.7 Reverse Transcription PCR (RT‐PCR) and Real‐Time Quantitative PCR (qPCR)

Total RNA was extracted from the striatum of mice using TRIzol reagent according to the manufacturer's protocol. Reverse transcription was carried out using the high‐capacity cDNA reverse transcriptase kit (Applied Biosystems) to synthesize cDNA. cDNAs were analysed by PCR using the following conditions: 95°C for 1 min, 60°C for 1 min and 72°C for 1 min (35 cycles). PCR products were separated by agarose gel electrophoresis and visualized using an Azure 300 imaging system (Azure Biosystems, CA, USA). qPCR was carried out with a QuantStudio 5 real‐time PCR system (Applied Biosystems, MA, USA). GAPDH was used as a reference gene. The sequences of primers were obtained from PrimerBank (Harvard University).

### 2.8 Western Blot

Western blot was performed as previously described [27]. Equal amounts of striatum lysates were mixed with denaturing 6x Laemmli loading dye and boiled for 5 min. Samples with equal amounts of total protein (50 μg per lane) were separated by 10% sodium dodecyl sulfate polyacrylamide gel, and proteins were transferred to a PVDF membrane (Bio‐Rad) using Trans‐Blot Turbo Transfer System (Bio‐Rad, USA). Membranes were blocked for 1 h at 21°C ± 1°C in Tris‐buffered saline with 0.1% Tween‐20 (TBST) (100 mmol/L NaCl, 10 mmol/L Tris, pH 7.4) and 5% nonfat milk followed by overnight incubation at 4°C with the following primary antibodies: anti‐D1DR (dopamine D1R) (Santa Cruz Biotechnology; sc‐33 660), anti‐D2DR (dopamine D2R) (Proteintech; 55 084–1‐AP), anti‐phospho‐Akt (Ser473) (Cell Signalling Technology; #9271), anti‐Akt (Cell Signalling Technology; #9272), anti‐CB1R (cannabinoid 1 receptor) (Proteintech; 17 978–1‐AP) and anti‐GAPDH (Santa Cruz Biotechnology; sc‐365 062). The dilutions of primary antibodies were 1:1000. Bound antibodies were detected with horseradish peroxidase‐conjugated antirabbit (cat: 31460) or antimouse (cat: 31430) antibodies (Thermo Scientific; diluted 1:10000) and visualized by Azure 300 imaging system (Azure Biosystems, CA, USA). The protein expressions were normalized to GAPDH.

### 2.9 Statistical Analysis

One‐way analysis of variance (ANOVA) with multiple comparisons using post hoc Tukey's test was used to compare the data from mice without receiving treatments. Two‐way ANOVA with multiple comparisons using post hoc Šidák's test was used to compare the data from mice treated with EtOH or saline. Student's *t*‐test (unpaired, two‐tailed) was also used. GraphPad Prism 10 (Boston, MA, USA) was used for analysis. Data are presented as the mean ± SEM. The value of *p* < 0.05 was considered significant.

## 3 Results

### 3.1 Generation of D2S KO Mice and Characterization of Motor‐ and Anxiety‐Related Behaviours

To generate D2S KO mice, we successfully performed genetic modifications so that D2L‐type mRNA containing exons 5, 6 and 7 is always expressed and mRNA lacking exon 6 is not expressed. In this way, D2S KO mice were generated by taking advantage of the new method using the artificial intron, RNA splicing and Cre‐loxP recombination [36]. The Drd2 (i.e., D2R) (ex5‐7)‐flox without Neo gene mice were designed to express both D2S and D2L isoforms. To produce D2S KO mice, the Drd2 (ex5‐7)‐flox without Neo gene mice were bred with Cre recombinase‐expressing mice to delete the region between loxP in intron 4 and loxP in intron 7. The resulting D2S KO mice expressed only the D2L isoform type mRNA containing exons 5, 6 and 7 following exon 4 (Figure 1A).

D2S KO mice have normal fertility, body weight and normal Mendelian genotypic ratio and look healthy similar to WT mice. mRNA expression levels of D2L/D2S in the striatum of D2S KO/D2L KO mice were assessed by RT‐PCR. In D2S KO mice, only D2L was expressed and the expression level of D2L was similar to the native D2R level in WT mice (Figure 1B). In D2L KO mice, only D2S was expressed (Figure 1B), which is consistent with our previous report [31]. To determine whether the expression levels of D1R and D2R were affected in D2S KO mice, the protein expression levels of D1R and D2R in three genotypes of mice were assessed by Western blot analysis. There were no significant differences in the protein expression levels of D1R and D2R between D2S KO, WT and D2L KO mice in the striatum (Figure 1C–E).

**FIGURE 2:**
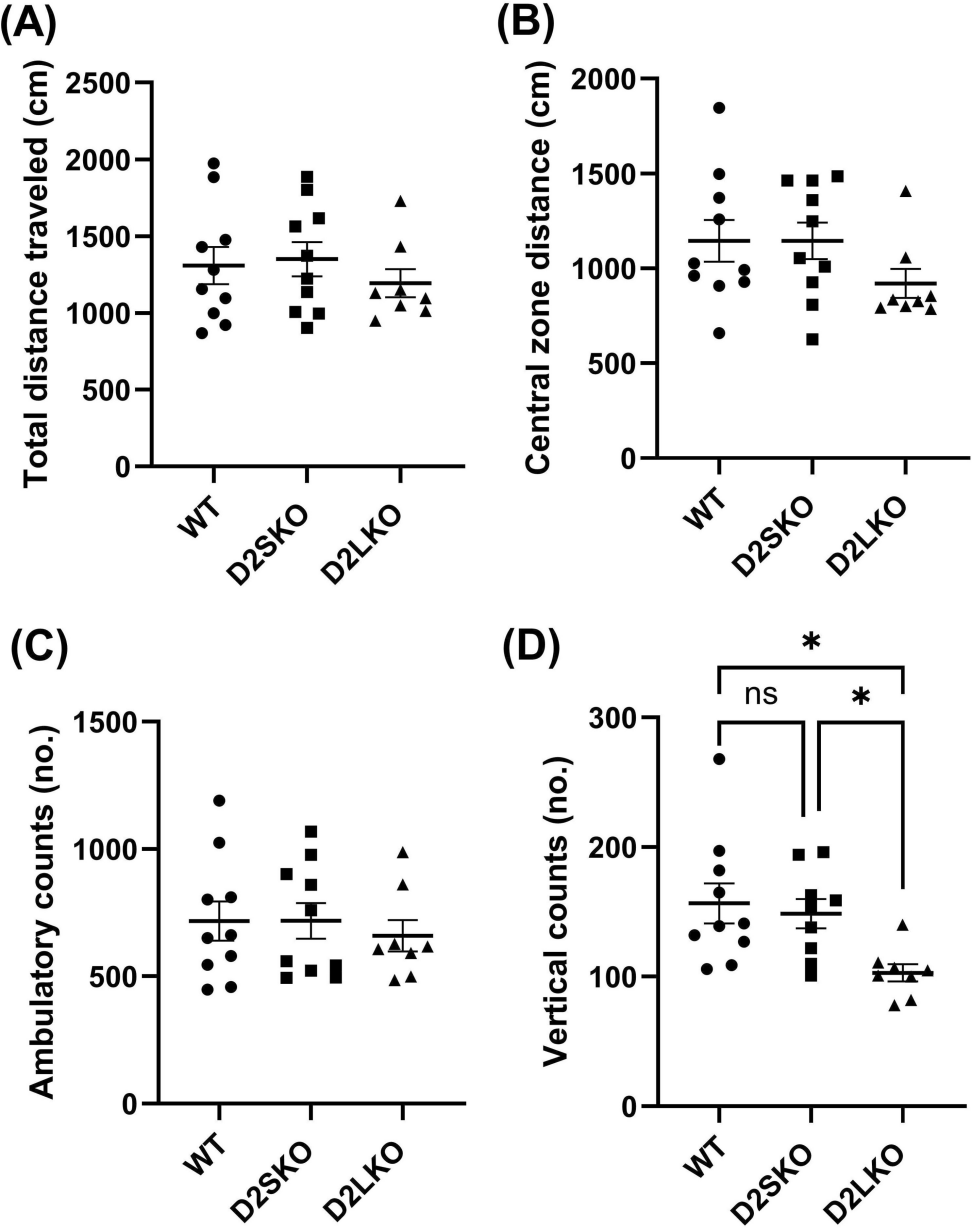
Basal locomotor activity of three genotypes of mice in the open‐field test. There were no statistical differences in total distance travelled (A) [*F*(2, 24) = 0.529; *p* = 0.5957; one‐way ANOVA with post hoc Tukey's test], distance travelled in central zone (B) [*F*(2, 24) = 1.582; *p* = 0.226] and ambulatory counts (C) [*F*(2, 24) = 0.276; *p* = 0.761] among WT, D2S KO and D2L KO mice. (D) The numbers (no.) of vertical counts (rearing behaviour) of D2L KO mice were significantly lower than those of WT and D2S KO mice [*F*(2, 24) = 5.127; *p = 0.014; one‐way ANOVA with post hoc Tukey's test]. WT, *n* = 10; D2SKO, *n* = 9; D2LKO, *n* = 8. ns, not significant.

**FIGURE 3:**
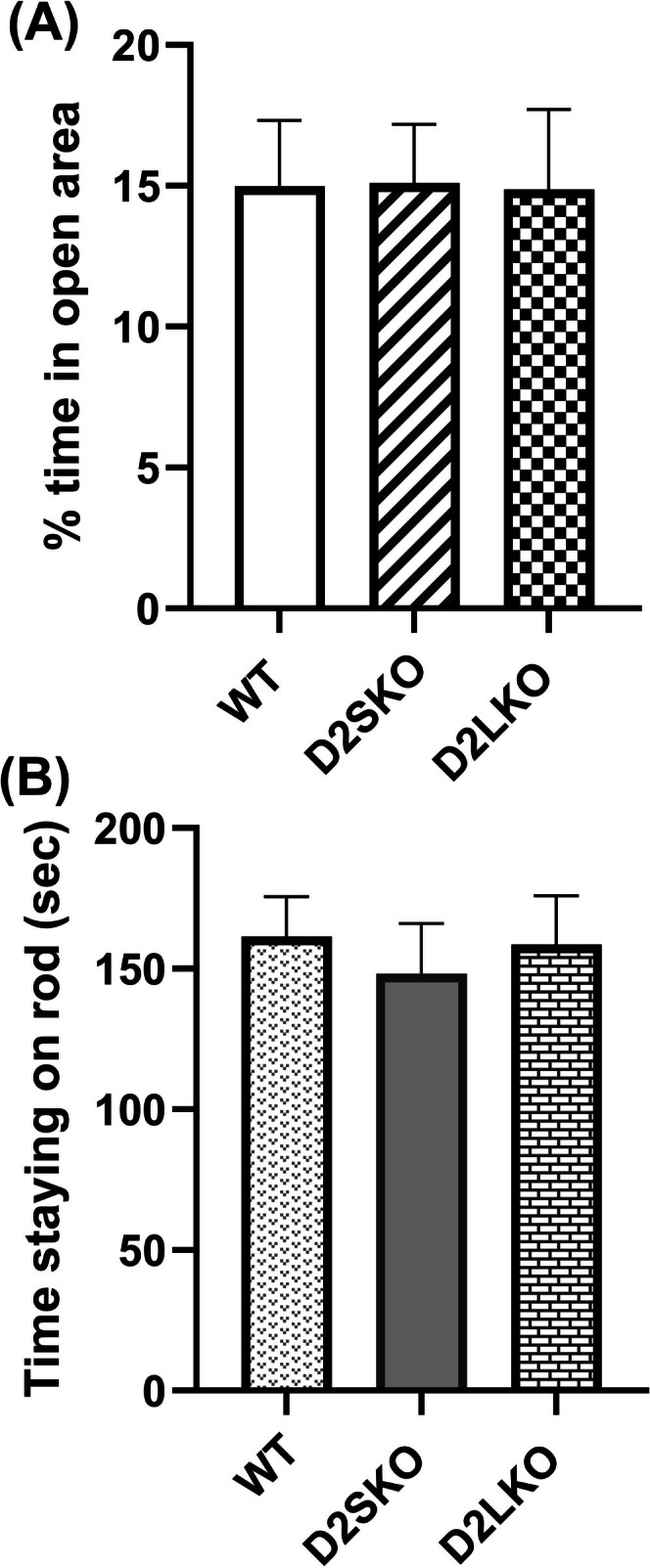
Anxiety status and motor coordination of three genotypes of mice. (A) Zero‐maze test. There was no significant difference in the percentage of time that mice spent in open sectors among the three genotypes of mice [*F*(2, 24) = 0.012; *p* = 0.988; one‐way ANOVA with post hoc Tukey's test]. (B) Rotarod test. There was no significant difference in time staying on the accelerating rod (sec) among the three genotypes of mice [*F*(2, 24) = 0.062; *p* = 0.939]. WT, *n* = 10; D2SKO, *n* = 9; D2LKO, *n* = 8.

Basal levels of locomotor activity, anxiety status and motor coordination were examined in D2S KO mice (expressing only D2L) as compared to D2L KO mice (expressing only D2S) and WT (in which D2L expression is much higher than D2S overall) [31, 40] using the open‐field, elevated zero‐maze and rotarod tests. The absence of D2S did not affect much basal locomotor activity of D2S KO mice including total distance travelled (Figure 2A), distance travelled in central zone (Figure 2B) and ambulatory counts (Figure 2C) as compared to WT. The basal locomotor activity of D2S KO mice was somewhat higher than that of D2L KO mice, but the differences did not reach significant. Also, there was no significant difference in vertical counts (rearing behaviour) between D2S KO and WT mice (*p* = 0.886) (Figure 2D). However, vertical counts of D2S KO and WT mice were significantly higher than those of D2L KO mice (*p* = 0.014) (Figure 2D). Furthermore, there was no significant difference in anxiety‐related behaviour among three genotypes of mice in the elevated zero‐maze test (*p* = 0.998) (Figure 3A), and there was no significant difference in basal levels of motor coordination among three genotypes of mice in the rotarod test (*p* = 0.829) (Figure 3B).

### 3.2 Alcohol‐Elicited CPP in D2L KO but Not D2S KO Mice

**FIGURE 4:**
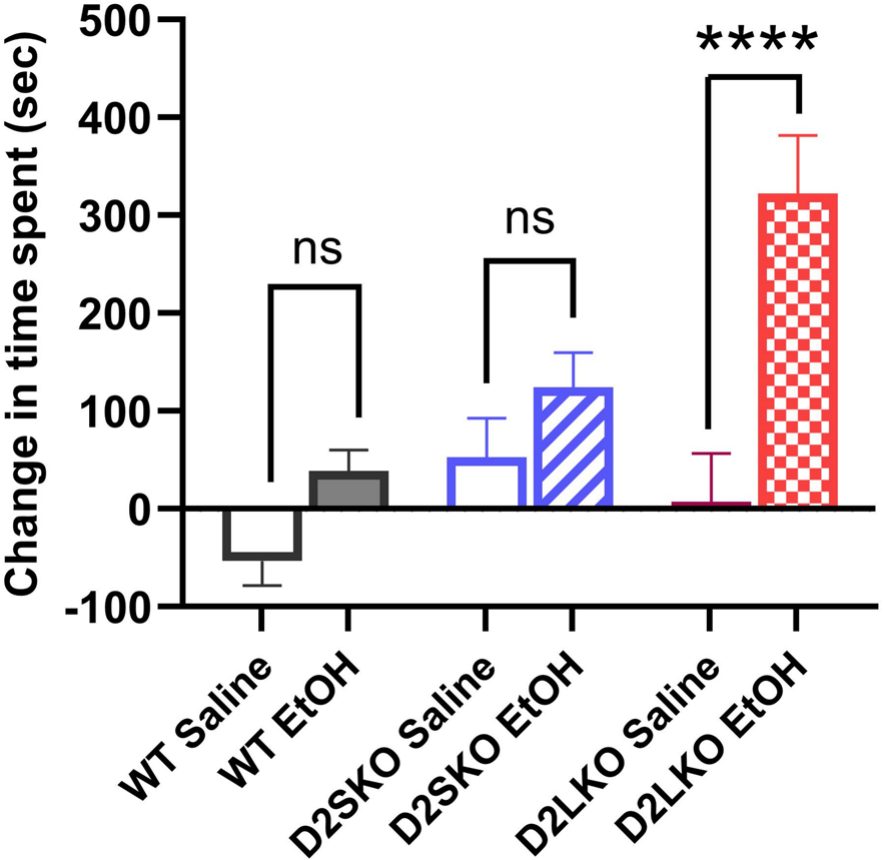
EtOH‐induced CPP was assessed in three genotypes of mice. The vertical bars represent the change in time spent (seconds) from preconditioning to postconditioning in EtOH‐paired compartment or saline‐paired compartment. CPP was significantly increased in D2L KO mice treated with EtOH (D2LKO EtOH) as compared to D2L KO mice treated with saline (D2LKO Saline) (****p < 0.0001; Šidák's multiple comparisons test following two‐way ANOVA). There were no significant changes in CPP between D2S KO mice treated with EtOH (D2SKO EtOH) and D2S KO mice treated with saline (D2SKO Saline) (*p* = 0.5664) and between WT mice treated with EtOH (WT EtOH) and WT treated with saline (WT Saline) (*p* = 0.3543). The interaction (genotype x treatment) is considered significant [*F*(2, 45) = 3.511, *p* = 0.0383; two‐way ANOVA]. Effect of treatment: *F*(1, 45) = 17.73, *p* = 0.0001. Effect of genotype: *F*(2, 45) = 6.556, *p* = 0.0032. No significant differences were found between saline‐treated groups (WT Saline vs. D2SKO Saline, *p* = 0.5901; WT Saline vs. D2LKO Saline, *p* = 0.9313; D2SKO Saline vs. D2LKO Saline, *p* = 9709). WT, *n* = 6 (saline) and 10 (EtOH); D2S KO, *n* = 8 (saline) and 8 (EtOH); D2L KO, *n* = 7 (saline) and 8 (EtOH). ns = not significant.

To determine whether D2L or D2S plays a role in alcohol reward or addiction, we conducted a CPP test in three genotypes of mice. As shown in Figure 4, D2L KO mice displayed a significant increase in preference for the compartment paired with EtOH administrations compared to D2L KO mice receiving only saline administrations (*p* < 0.0001). On the other hand, no significant changes were observed in time spent in the compartment paired with EtOH administrations in D2S KO mice (*p* = 0.5664) and in WT mice (*p* = 0.3543) compared to their respective saline controls. These results suggest that the D2S isoform plays a bigger role than D2L in alcohol‐induced reward.

### 3.3 Motor Coordination Ability of D2L KO Mice Might Be More Susceptible to Alcohol Treatment

**FIGURE 5:**
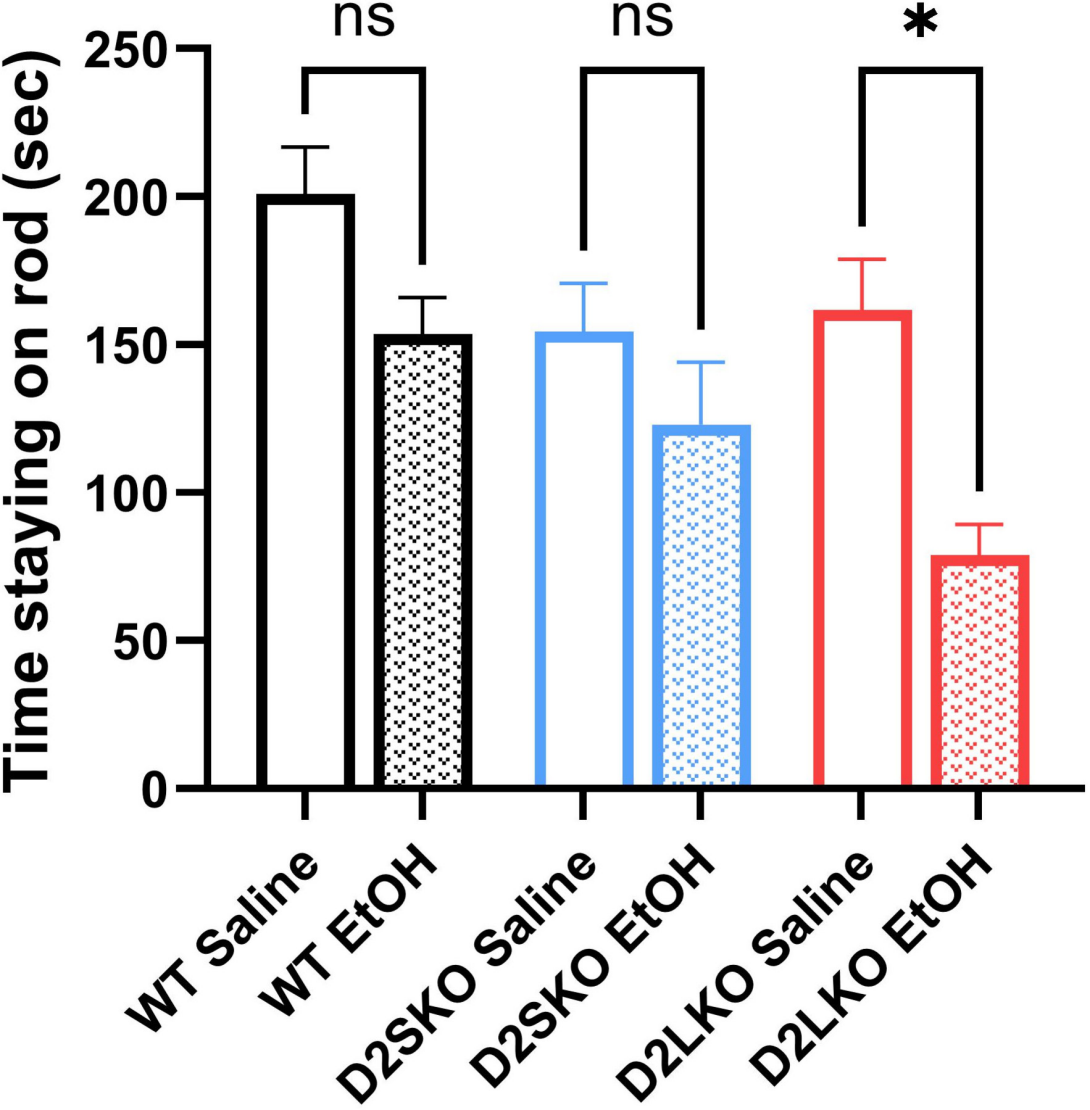
Rotarod performance of WT, D2S KO and D2L KO mice after chronic EtOH exposure. EtOH exposure significantly reduced the time spent on the accelerating rod in the D2LKO EtOH group as compared to the D2LKO saline group (*p = 0.034; Šidák's multiple comparisons test following two‐way ANOVA). There were no significant changes in Rotarod performance between D2SKO EtOH and D2SKO Saline (*p* = 0.473) and between WT EtOH and WT Saline (*p* = 0.074). No significant differences were found between the saline groups: WT Saline vs. D2SKO Saline (*p* = 0.201); WT Saline vs. D2LKO Saline (*p* = 0.171); D2SKO saline vs. D2LKO saline (*p* = 0.988). The interaction (genotype x treatment) is not significant (*F*(2, 39) = 0.7488, *p* = 0.4796, two‐way ANOVA). But both effect of treatment and effect of genotype are significance. Effect of treatment: *F*(1, 39) = 13.63, *p* = 0.0007. Effect of genotype: *F*(2, 39) = 3.747, *p* = 0.0325. WT, *n* = 6 (saline) and 7 (EtOH), D2SKO, *n* = 9 (saline) and 9 (EtOH), D2LKO, *n* = 6 (saline) and 7 (EtOH). ns = not significant.

To determine the influence of individual D2R isoforms on alcohol‐induced changes in gene expression, three genotypes of mice were treated with EtOH (or saline) for 21 days. Before gene analysis, we examined the motor coordination of these mice using the rotarod test, as a previous study has reported that alcohol has detrimental effects on motor coordination [41]. A longer time spent on the accelerating rod is indicative of better motor coordination. EtOH‐treated D2L KO mice performed significantly worse on the accelerating rod than saline‐treated D2L KO mice (*p* = 0.034) (Figure 5). On the other hand, there were no significant differences in motor coordination between EtOH‐treated D2S KO mice and saline‐treated D2S KO mice (*p* = 0.473) and between EtOH‐treated WT mice and saline‐treated WT mice (*p* = 0.074) (Figure 5). No significant differences were found between the saline groups (Figure 5).

### 3.4 Chronic Alcohol Exposure Altered the Expression of Cannabinoid Receptors in D2L KO but Not D2S KO Mice

To determine the molecular consequences of increased D2S to D2L expression that led to alcohol CPP, we examined the expression of several receptors/genes that have been shown to interact with the D2R system and/or be involved in AUD. We first assessed the expression levels of CB1R and CB2R in three genotypes of mice chronically treated with EtOH as compared to mice treated with saline. Proteins or mRNAs were isolated from the striatum of mice that received either EtOH or saline administrations for 21 days.

**FIGURE 6:**
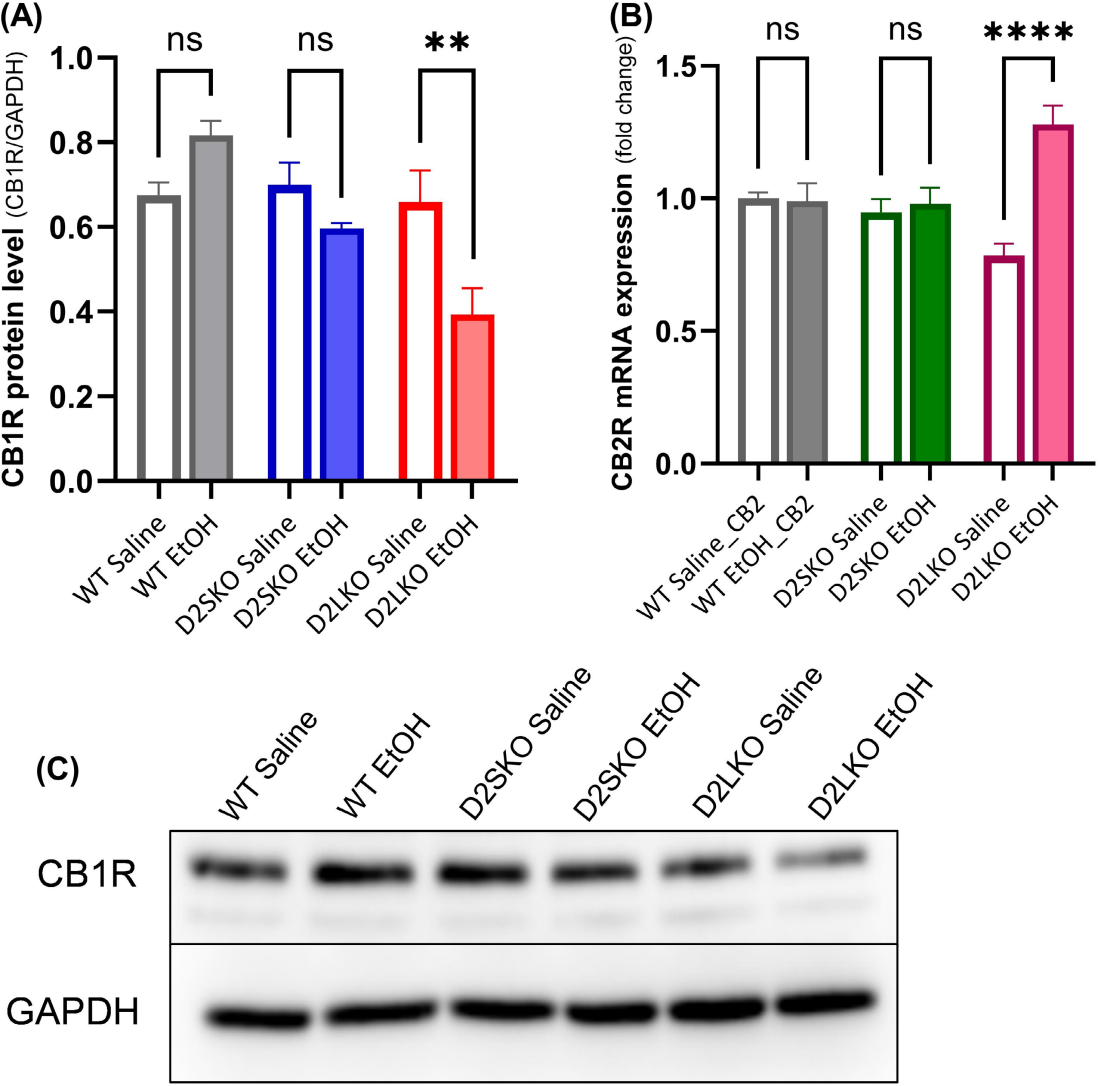
Western blot and qPCR analysis of cannabinoid receptor expression in the striatum after chronic alcohol exposure. Mice received EtOH or saline injections for 21 days. (A) The relative protein expression levels of CB1R in the striatum of WT, D2S KO and D2L KO mice treated with EtOH or saline were analysed by Western blotting (WB) shown in (C). Protein quantification was normalized using GAPDH. A significant decrease was observed in D2LKO EtOH group as compared to D2LKO Saline group (**p = 0.0038; Šidák's multiple comparisons test following two‐way ANOVA). WT EtOH vs. WT Saline (*p* = 0.1727). D2SKO EtOH vs. D2SKO Saline (*p* = 0.4096). The interaction (genotype x treatment) is considered significant [*F*(2, 18) = 8.543, *p* = 0.0025; two‐way ANOVA]. Effect of treatment: *F*(1, 18) = 3.585, *p* = 0.0745. Effect of genotype: F(2.18) = 9.760, *p* = 0.0013. (B) The relative expression levels of CB2R mRNA in the striatum of WT, D2S KO and D2L KO mice treated with EtOH or saline were analysed by qPCR. A significant increase was observed in D2LKO EtOH group as compared to D2LKO Saline group (****p < 0.0001; Šidák's multiple comparisons test following two‐way ANOVA). WT EtOH vs. WT Saline (*p* = 0.9977). D2SKO EtOH vs. D2SKO Saline (*p* = 0.9680). The interaction (genotype x treatment) is considered significant [*F*(2, 66) = 13.05, *p* < 0.0001; two‐way ANOVA]. Effect of treatment: *F*(1, 66) = 14.53, *p* = 0.0003. Effect of genotype: F(2.66) = 0.7759, *p* = 0.4645. GAPDH was used as a reference gene. The values are the means (± SE) of four independent experiments for WB and the means (± SE) of four independent experiments for qPCR experiment with three replicates per sample per experiment. ns = not significant.

A significant decrease in the protein expression levels of CB1R was observed in EtOH‐treated D2L KO mice compared to saline‐treated D2L KO mice (*p* = 0.0038) (Figure 6A,C). No significant changes in CB1R protein levels were observed in EtOH‐treated WT mice (*p* = 0.1727) and EtOH‐treated D2S KO mice (*p* = 0.4096) compared to their respective saline control groups (Figure 6A,C). On the other hand, a significant increase in the mRNA expression levels of CB2R was observed in EtOH‐treated D2L KO mice compared to saline‐treated D2L KO mice (*p* < 0.0001) (Figure 6B). No significant changes in CB2R mRNA levels were observed in EtOH‐treated WT mice (*p* = 0.9977) and EtOH‐treated D2S KO mice (*p* = 0.9680) as compared to their respective saline control groups. These results suggest that an increased expression level of D2S to D2L (here D2L is deficiency with concomitant D2S overexpression) affects the expression of CB1R and CB2R in the striatum of mice following chronic alcohol exposure.

### 3.5 Chronic Alcohol Exposure Altered Akt Phosphorylation in D2L KO but Not D2S KO Mice

It has been shown that Akt is a downstream target of the D2R signalling and can be modulated by cannabinoids [26, 27]. We then examined the levels of Akt and Akt phosphorylation at Ser473 (p‐Akt) in the striatum of WT and two D2R isoform KO mice after chronic alcohol administration.

**FIGURE 7:**
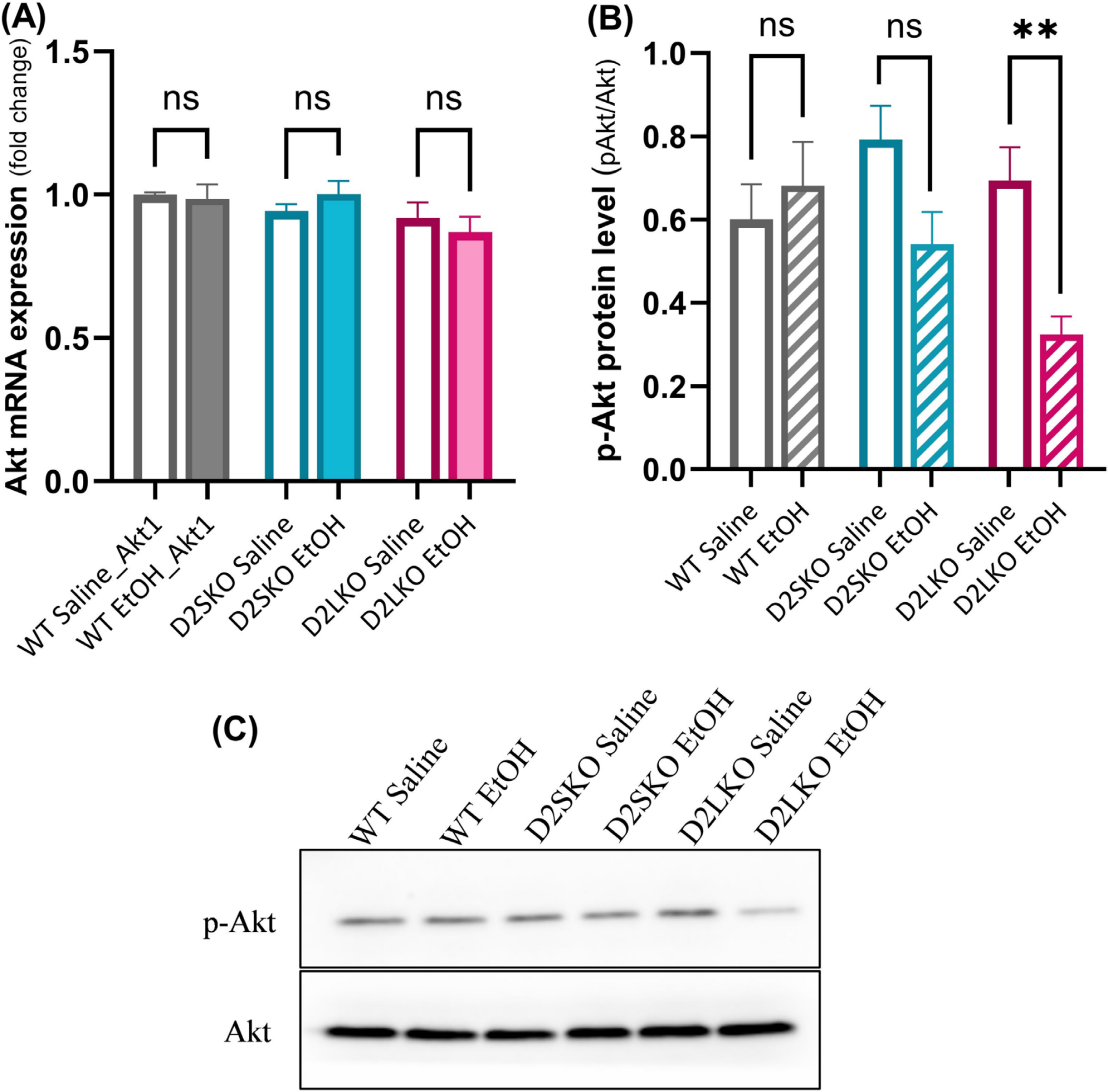
qPCR and Western blot analysis of Akt and p‐Akt expression after chronic alcohol exposure. (A) The relative expression levels of Akt1 mRNA in the striatum of WT, D2S KO and D2L KO mice treated with EtOH or saline were analysed by qPCR. There were no significant changes in Akt1 mRNA expression levels between saline and EtOH in WT, D2SKO and D2L KO groups [*F*(2, 66) = 0.825, *p* = 0.4442; two‐way ANOVA). WT Saline vs. WT EtOH (*p* = 0.9940; post hoc Šidák's test), D2SKO Saline vs. D2SKO EtOH (*p* = 0.7159) and D2LKO Saline vs. D2LKO EtOH (*p* = 0.7976). (B) Relative protein expression levels of p‐Akt at Ser473 in the striatum of WT, D2S KO and D2L KO mice treated with EtOH or saline were analysed by Western blotting (WB). Significant decrease was observed in D2LKO EtOH group compared to D2LKO saline group (**p = 0.0090, post hoc Šidák's test following two‐way ANOVA). WT EtOH vs. WT Saline (*p* = 0.8605). D2SKO EtOH vs. D2SKO Saline (*p* = 0.1005). The interaction (genotype x treatment) is considered significant [*F*(2, 30) = 4.191, *p* = 0.0248; two‐way ANOVA]. Effect of treatment: *F*(1, 30) = 7.455, *p* = 0.0105. Effect of genotype: (2, 30) = 2.169, *p* = 0.1319. (C) Western blots of p‐Akt at Ser473 and Akt (as control) in mouse striatum. The value of p‐Akt was normalized to the amount of total Akt and expressed as a percentage of control treatment. No significant differences in Akt protein levels were observed between any genotype pairs (see Supplemental Materials). The values are the means (± SE) of four independent experiments for WB and the means (± SE) of four independent experiments for qPCR experiment with three replicates per sample per experiment.

The mRNA expression levels of Akt1 were not significantly altered in the striatum of D2L KO, D2S KO and WT mice treated with EtOH as compared to their respective saline controls (*p* = 0.7976, *p* = 0.7159 and *p* = 0.9940, respectively] (Figure 7A). Also, no significant change in Akt protein levels was observed among three genotypes of mice (Figure 7C; also see Supporting Information). On the other hand, the protein level of p‐Akt (Ser473) was significantly decreased in the striatum of EtOH‐treated D2L KO mice compared to saline‐treated D2L KO mice (*p* = 0.0090) (Figure 7B,C). There were no significant changes in p‐Akt levels between EtOH‐treated WT mice and their saline controls (*p* = 0.8605) and between EtOH‐treated D2S KO mice and their saline controls (*p* = 0.1005) (Figure 7B,C). These results highlight the importance of a balance in D2S to D2L ratio in modulating Akt phosphorylation in mice with chronic exposure to alcohol.

### 3.6 Chronic Alcohol Exposure Reduced Tyrosine Hydroxylase Expression in D2L KO but Not D2S KO Mice

**FIGURE 8:**
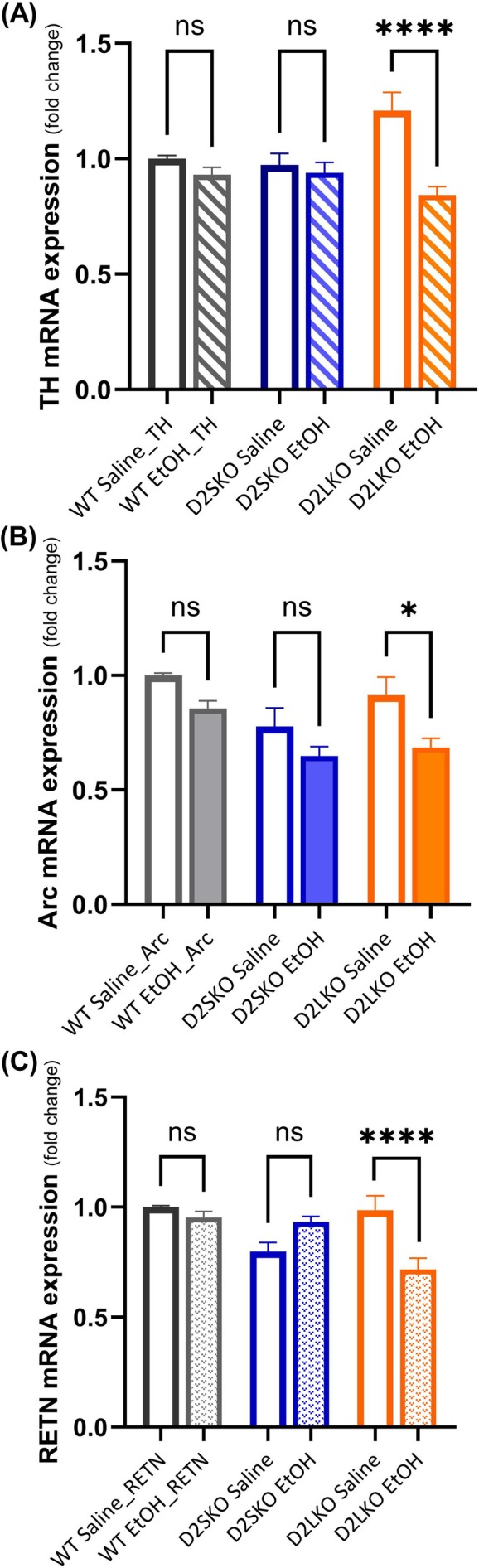
The expressions of TH, Arc and RETN mRNAs in the striatum after chronic alcohol exposure were quantified by qPCR. The y‐axis indicates the relative mRNA expression level of the gene. (A) TH expression was significantly decreased only in D2L KO mice treated with EtOH (D2LKO EtOH) as compared to D2L KO mice treated with saline (D2LKO Saline) (****p < 0.0001; post hoc Šidák's test following two‐way ANOVA). WT EtOH vs. WT Saline (*p* = 0.6513). D2SKO EtOH vs. D2SKO Saline (*p* = 0.9410). The interaction (genotype x treatment) is considered significant [*F*(2, 66) = 7.559, *p* = 0.0011; two‐way ANOVA]. Effect of treatment: *F*(1, 66) = 16.74, *p* = 0.0001. Effect of genotype: F(2.66) = 0.2748, *p* = 0.2748. (B) Arc expression was significantly decreased in D2LKO EtOH group compared to D2LKO Saline group (*p = 0.0108; post hoc Šidák's test following two‐way ANOVA). D2SKO EtOH vs. D2SKO Saline (*p* = 0.2626). WT EtOH vs. WT Saline (*p* = 0.1714). The interaction is not significant [*F*(2, 66) = 0.5100, *p* = 0.6028, two‐way ANOVA]. But both effect of treatment and effect of genotype are significant. Effect of treatment: *F*(1, 66) = 14.57, *p* = 0.0003; Effect of genotype: *F*(2, 66) = 8.029, *p* = 0.0008. (C) RETN expression was significantly decreased only in D2LKO EtOH group as compared to D2LKO Saline group (****p < 0.0001; post hoc Šidák's test following two‐way ANOVA). WT EtOH vs. WT Saline (*p* = 0.8092). D2SKO EtOH vs. D2SKO Saline (*p* = 0.0734). The interaction is considered significant [*F*(2, 66) = 11.92, *p* < 0.0001; two‐way ANOVA]. Effect of treatment: *F*(1, 66) = 3.231, *p* = 0.0768. Effect of genotype: *F*(2, 66) = 5.472, *p* = 0.0063. ns = not significant. GAPDH was used as a reference gene. The expression level of each gene was normalized to that of GAPDH and compared to WT Saline values. The values are the means (± SE) of four independent experiments with three replicates per sample per experiment.

Chronic alcohol consumption can cause altered DA release in the striatum [13]. Tyrosine hydroxylase (TH) is a key enzyme in DA synthesis. To explore if either D2R isoform is involved in regulating TH expression in alcohol use, we examined TH mRNA expression in the striatum of three genotypes of mice after chronic EtOH administration. As shown in Figure 8A, the mRNA expression level of TH was significantly reduced in EtOH‐treated D2L KO mice compared to saline‐treated D2L KO mice (*p* < 0.0001). There were no significant changes in the TH expression level between EtOH‐treated WT mice and their saline controls (*p* = 0.6513) and between EtOH‐treated D2S KO mice and their saline controls (*p* = 0.9410). These results suggest that D2S may play a bigger role than D2L in regulating TH expression under alcohol influence.

### 3.7 D2L KO Mice Had Reduced Expression of Arc and RETN Following Chronic Alcohol Exposure

We further examined some selected genes of interest that are implicated in AUD to determine if altered expression of D2R isoforms influence the expression of these genes. We found two additional genes, Arc and RETN, showed expression changes mainly in EtOH‐treated D2L KO mice. The mRNA expression level of Arc in the striatum was significantly reduced in EtOH‐treated D2L KO mice compared to saline‐treated D2L KO mice (*p* = 0.0108) (Figure 8B). No significant changes in Arc expression were observed in EtOH‐treated D2S KO mice compared to their saline controls (*p* = 0.2626) and in EtOH‐treated WT mice compared to their saline controls (*p* = 0.1714). The mRNA expression level of RETN in the striatum was significantly reduced in EtOH‐treated D2L KO mice compared to saline‐treated D2L KO mice (*p* < 0.0001) (Figure 8C). There were no significant changes in the RETN expression level between EtOH‐treated WT mice and their saline controls (*p* = 0.8092) and between EtOH‐treated D2S KO mice and their saline controls (*p* = 0.0734).

## 4 Discussion

In the present study, we reported a new conditional KO mouse that is selectively deficient in D2S but still expresses D2L (i.e., D2S KO mice). Previous studies have reported that D2R null mice (which lack both D2L and D2S) show abnormal growth, fertility, body weight, colour and a skewed Mendelian genotypic ratio [42, 43, 44]. In contrast, D2S KO mice described here have normal growth, fertility, colour and body weight similar to WT mice and have a normal genotypic Mendelian ratio. qPCR analysis showed that D2L was the only D2R isoform expressed in D2S KO mice and expressed at a level similar to total D2R level in WT mice. In addition, the protein expression levels of D1R and D2R in D2S KO mice were comparable to those of WT and D2L KO mice. Furthermore, in several analyses that assessed brain functions related to D2R signalling such as locomotor activity, rearing behaviour, motor coordination and alcohol CPP, the behaviours of D2S KO mice were similar or close to those of WT mice (in which D2L expression is much higher than D2S overall) [31, 40], suggesting that D2L in D2S KO mice is functional.

Note that the two D2R isoforms are generated by alternative splicing from the same gene. Our manipulation of the D2R genomic gene eliminated the possibility of transcription of one of the D2R isoforms, and thereby, the D2R gene was exclusively available for making another D2R isoform. This results in a compensatory increase in the isoform left expressing at a level similar to the total D2R level of WT mice.

Previous studies have reported that D2R null mice show reduced locomotor activity and rearing behaviour compared to WT mice [42, 44]. In contrast, the locomotor activity and rearing behaviour of D2S KO mice reported in this study were comparable to those of WT mice. The rearing behaviour of D2L KO mice were significantly reduced compared to D2S KO and WT mice, which is consistent with our previous findings [31]. In addition, the motor coordination ability of D2L KO mice appeared to be more susceptible to alcohol treatment than that of D2S KO and WT mice. These results provide further evidence supporting our previous suggestion that D2L plays a bigger role than D2S in regulating motor functions [31].

CPP has been used extensively to study the rewarding effects of drugs or substances [45]. A previous study has reported that D2R null mice (lacking both D2R isoforms) show a marked decrease in alcohol preference and are aversive to alcohol [46]. In the present study, we demonstrated that D2L KO mice displayed a strong alcohol‐induced CPP. On the other hand, D2S KO and WT mice did not exhibit significant alcohol CPP, but were not aversive to alcohol either. These results suggest that an increased expression level of D2S to D2L (e.g., D2L deficiency with concomitant D2S overexpression) may be an underlying mechanism mediating the positive reinforcement of alcohol.

The purpose of the CPP experiment was to examine the sensitivity of three genotypes of mice to the EtOH‐induced rewarding effect. The CPP protocol used was moderate (e.g., only four EtOH injections). Some of the protocols used by others are more vigorous or the strains of mice used are more sensitive to the EtOH effect (e.g., C57BL/6 J mice are more sensitive to EtOH effects than C57BL/6 N). When analyzing the data by Student's *t‐*test (unpaired), CPP was also significantly increased in EtOH‐treated WT mice compared to saline‐treated WT (*p* = 0.019; t‐test), whereas there was still no significant difference in CPP between EtOH‐treated D2SKO mice and saline‐treated D2SKO mice (*p* = 0.201; *t*‐test) (see Figure S4). This implies that WT mice were more sensitive to the EtOH‐induced rewarding effect than D2SKO mice, although to a lesser extent than D2LKO mice (*p* = 0.001; *t*‐test).

Previously, we reported that D2L KO mice displayed a deficit in morphine CPP, while retaining a similar level of cocaine CPP to that of WT mice [39]. In the present study, D2L KO mice developed a relatively strong alcohol CPP. These results together indicate that alterations in D2S to D2L ratio have differential effects on the rewarding effects induced by morphine, cocaine, or alcohol, respectively. Previous studies have also shown that changes in the level of one of the D2R isoforms are associated with substance use disorder. Moyer et al. [47] have found that polymorphisms in introns 5 and 6 in human postmortem prefrontal cortex and striatum from cocaine addicts cause reduced D2S expression relative to D2L. Giordano III et al. [48] have reported that in amphetamine‐sensitized mice, D2L mRNA but not D2S mRNA is upregulated in the striatum. Although most abused substances can induce DA release in the striatum, the underlying mechanisms of how these substances elicit reward are complex and multifactorial. Gallo et al. [49] have shown that genetically increasing D2RL (the long isoform) expression in the NAc does not cause an increase in alcohol consumption in mice compared to controls. We previously demonstrated that D2L KO mice (with D2S overexpression) displayed excessive EtOH drinking behaviour compared to WT mice [50]. Our study implies the possibility that an alteration in the expression of D2S vs. D2L could be one of the factors contributing to the development of AUD. There is evidence suggesting that abnormal alternative splicing is implicated in the pathophysiology of several neurodegenerative diseases [51].

Growing evidence suggests that endocannabinoid receptors (CB1R and CB2R) play a role in AUD and CB1R expression in the cortex, and striatum can be modulated by D2R and chronic alcohol consumption [23]. Following chronic alcohol administration, the mRNA expression of the CB1R in different brain regions, including the striatum, has been shown to either decrease or increase in rats [52, 53]. Preclinical studies in rodents have shown that CB1R agonists increase alcohol consumption [54], while a CB1R antagonist reduces alcohol preference and craving [55]. The reported roles of CB2R in alcohol‐mediated effects are various. It has been shown that a CB2R agonist enhances alcohol preference, while a CB2R antagonist blocks alcohol preference in mice [56]. Conversely, CB2R KO mice show increased alcohol consumption and preference [57]. Clinical investigations have found polymorphisms in CB1R and CB2R genes in patients with AUD [56].

In the present study, we found that chronic alcohol administration caused differential changes in the expressions of CB1R and CB2R—CB1R expression was downregulated, while CB2R expression was upregulated in the striatum of EtOH‐treated D2L KO mice. These changes were absent in D2S KO and WT mice. This study provides further evidence indicating both CB1R and CB2R are involved in alcohol‐induced effects. In addition, our results suggest that a higher D2S to D2L expression ratio (e.g., in the case of D2L deficiency with concomitant D2S overexpression) can have a significant impact on the expression of both CB1R and CB2R in response to alcohol exposure in the striatum, a brain region that is involved in the rewarding circuits and habit formation and plays a crucial role in drug addiction [58, 59].

It has been shown that Akt is one of the main downstream effectors affected by CB1R activity and activation of CB1R increases the phosphorylation of Akt (p‐Akt) in the striatum and other brain areas [27, 60]. Interestingly, Akt is also a downstream target of the D2R signalling [26]. Research has identified that the Akt signalling pathway is involved in alcohol‐induced effects in the brain, although the results are mixed; either decreased or increased p‐Akt expression is observed in the ventral or dorsal striatum of mice or rats after alcohol exposure [61, 62]. We found that although there was no significant change in the mRNA and protein expression levels of Akt among three genotypes of the mice, the p‐Akt (ser 473) level was reduced selectively in EtOH‐treated D2L KO group. No significant change in p‐Akt levels was observed in EtOH‐treated D2S KO and WT mice. These results suggest that the increased D2S to D2L expression level may cause alterations in the cannabinoid‐Akt signalling pathway, which may be a significant factor contributing to the development of alcohol‐induced reinforcement and compulsive drinking.

The role of DA in AUD is well‐documented. The expression of TH, a rate‐limiting enzyme in DA biosynthesis, has been shown to be reduced in the ventral striatum after chronic alcohol exposure [63]. Interestingly, cannabinoids have been shown to alter dopaminergic transmission by modulating TH activity. Cannabinoid agonists can increase TH expression in the striatum [64]. Another study has shown that cannabidiol can reduce EtOH consumption and downregulate TH gene expression in the ventral tegmental area (VTA) [65]. In this study, we observed that TH mRNA expression was significantly decreased only in EtOH‐exposed D2L KO mice (expressing only D2S) but not in D2S KO mice (expressing only D2L) and WT mice (expressing mostly D2L). These results suggest that D2S may play a bigger role than D2L in the regulation of TH expression in response to chronic alcohol exposure. A decrease in TH expression would result in a reduction in DA production and consequently affect DA receptor‐mediated signalling and behaviours. It has been reported that D2S (but not D2L) plays a role in regulating TH activity in nigrostriatal presynaptic sites [66].

Previous studies have shown that inhibiting Arc expression increases alcohol consumption in rats [67], and Arc KO mice exhibit a higher alcohol addiction score than WT mice [68]. Decreased phosphorylated Akt and reduced TH activity have been observed in the striatum of Arc KO mice [29]. In this study, the multiple comparisons test indicated that Arc mRNA expression levels were significantly decreased only in EtOH‐exposed D2L KO mice compared to their respective saline‐exposed groups. In contrast, no significant differences were observed in EtOH‐exposed D2S KO mice and EtOH‐exposed WT mice. Based on data analysis, there is a possibility that chronic alcohol exposure may affect the expression of Arc, a synaptic plasticity‐related gene, in D2L KO mice. Saline‐treated D2S KO mice had lower basal Arc expression than saline‐treated D2L KO mice and saline‐treated WT mice; there is a possibility that D2L overexpression may influence basal Arc expression. However, the EtOH effect on Arc expression was not significant in D2S KO mice.

RETN, the gene encoding resistin, has been shown to be involved in alcohol craving or dependence in humans [69, 70]. In this study, we found that RETN was significantly downregulated in the striatum of D2L KO mice chronically exposed to alcohol, whereas there were no significant changes in RETN levels in D2S KO and WT mice. Our study provided evidence for the first time showing that the RETN expression could be modulated by the D2R system. These results suggest that the increased expression of D2S to D2L plays a significant role in modulating alcohol effects on the expression of RETN.

Our results showed that a higher D2L to D2S expression made a difference in attenuating alcohol‐induced impairment of motor coordination as displayed by D2S KO mice. However, the increased expression of D2L to D2S has little effect on alcohol‐induced CPP and signalling changes with respect to the genes examined in this study. This is probably because we focused on genes that are implicated in AUD in this study. A higher D2L/D2S ratio may affect a different set of genes involved in other functions.

Are the behavioural and molecular phenotypes observed in D2L KO mice due to the lack of D2L or the overexpression of D2S? Our results suggest that the phenotypic changes shown in the present study are likely to result from the upregulated expression of D2S. As described in the discussion earlier, Phillips and colleagues [46] have reported that D2R null mice (lacking both D2L and D2S) show a marked decrease in alcohol preference and are aversive to alcohol. The present study showed that D2L KO mice (D2L deficiency with concomitant D2S overexpression) displayed a strong alcohol‐induced CPP. The fact that mice lacking D2L displayed a strong EtOH CPP suggested that the enhanced positive reinforcement of alcohol displayed in D2L KO mice was likely attributed to the increased expression of D2S; otherwise, the reverse behavioural phenotype would be predicted. We then assessed the expression of several genes and proteins that are known to interact with the D2R system and play a role in AUD to decipher the potential mechanisms underlying the enhanced EtOH CPP displayed in D2L KO mice. It has been reported that in D2R null mice, CB1R is upregulated in the striatum [23]. If the change in D2L KO mice were due to the loss of D2L, CB1R would be upregulated in D2L KO mice. However, our results showed that CB1R was downregulated, suggesting that the EtOH effect was likely mediated by increased D2S expression. Similarly, in other experimental results, if the EtOH effects seen in D2L KO mice were due to the lack of D2L, then D2S KO mice (with overexpression of D2L) would exhibit opposite phenotypes from those of D2L KO mice. We cannot completely exclude the influence of D2L deficiency. Thus, we think it is more appropriate to interpret the results from a D2S/D2L expression alteration point of view.

## 5 Conclusion

The findings in this study shed light on the differential role of individual D2R isoforms in alcohol‐induced changes in behaviours and gene expression. Our results revealed that an increase in the expression level of D2S to D2L was associated with increased alcohol reinforcement and triggered a cascade of changes in the cannabinoid receptor and several gene systems following chronic alcohol exposure. These signalling changes as a consequence of the altered D2S/D2L expression may contribute to increased alcohol reward and consumption displayed in D2L KO mice. These findings suggest the altered splicing of D2R may have a significant impact on regulating some other receptor/signalling systems implicated in the brain reward mechanism. It is possible that a higher D2S/D2L ratio makes certain receptors/intracellular molecules more susceptible to alcohol exposure and is a risk factor leading to alcohol abuse and addiction. Our findings suggest that alterations in D2R splicing may represent a pathophysiological mechanism underlying the development of AUD and provide insights for discovering new treatment targets.

## Author Contributions

**Mohd Tayyab:** investigation, methodology, data curation, formal analysis, visualization, writing – original draft and review and editing. **Toshikuni Sasaoka, Manabu Abe, Nae Saito, Kenji Sakimura: Resource. Toshikuni Sasaoka, Manabu Abe, Kenji Sakimura:** funding acquisition. **Toshikuni Sasaoka:** writing – review and editing. **Anetta Ninan:** data curation, writing – review and editing. **YanYan Wang:** conceptualization, methodology, formal analysis, project administration, resources, supervision, funding acquisition, visualization, writing – original draft and review and editing.

## Ethics Statement

This study was carried out under approved protocol by the Institutional Animal Care and Use Committees at the University of Texas at Tyler Health Science Center and conducted in accordance with the National Institute of Health Guide for the Care and Use of Laboratory.

## Conflicts of Interest

The authors declare no conflicts of interest.

## Supporting information


**Figure S1:** The sequence of the artificial intron, the combined exons 5–6‐7 and the surrounding regions included in the targeting vector. The sequences from 70 bp at the 3′ end of intron 4 of the mouse Drd2 (D2R) gene to 85 bp of the 3′ downstream of the combined exons 5–6‐7 used in the targeting vector are described. The sequences of exons 5, 6 and 7 are written in blue, orange and green colour, respectively. The artificial intron is located between the downstream of exon 7 and the upstream of the combined exons 5–6‐7. The loxP sequence is written in red colour and the adenosine enclosed by a navy‐blue box represents an RNA splicing branch site.

**Table S1:** List of primary and secondary antibodies used in the study.
SFig. 4. EtOH‐induced CPP was assessed in three genotypes of mice. These are the same data used for figure 4 in the main text except that the statistical analysis was done using Student's *t‐*test (unpaired). CPP was significantly increased in EtOH‐treated D2L KO mice (D2LKO EtOH) as compared to saline‐treated D2L KO mice (D2LKO Saline) (**p = 0.001; *t‐*test, unpaired). CPP was also significantly increased in EtOH‐treated WT mice (WT EtOH) as compared to saline‐treated WT (WT Saline) (*p = 0.019; *t‐*test, unpaired). There was no significant change in CPP between EtOH‐treated D2S KO mice (D2SKO EtOH) and saline‐treated D2S KO mice (D2SKO Saline) (*p* = 0.201; *t‐*test, unpaired; ns = not significant).
SFig. 7. Relative protein expression levels of Akt in the striatum of WT, D2S KO, and D2L KO mice treated with EtOH or saline were analysed by Western blotting. No significant differences in Akt protein levels were observed between any genotype pairs. [WT Saline vs. WT EtOH, *p* = 0.7593; D2SKO Saline vs. D2SKO EtOH, *p* = 0.9900; D2LKO Saline vs. D2LKO EtOH, *p* = 0.6074; overall, *F*(2, 18) = 0.5788, *p* = 0.5707, two‐way ANOVA with post hoc Šidák's test].

## Data Availability

Data will be available on request from Y.W.
