# Supplementary material for: Dopamine D2S/D2L Receptor Regulation of Alcohol‐Induced Reward and Signalling
Source: Addict Biol. 2025 Nov 15;30(11):e70093. doi: 10.1111/adb.70093 (PMC12619067; doi:10.1111/adb.70093)
Supplement: Supplementary file 1 — Figure S1: The sequence of the artificial intron, the combined exons 5–6‐7 and the surrounding regions included in the targeting vector. The sequences from 70 bp at the 3′ end of intron 4 of the mouse Drd2 (D2R) gene to 85 bp of the 3′ downstream of the combined exons 5–6‐7 used in the targeting vector are described. The sequences of exons 5, 6 and 7 are written in blue, orange and green colour, respectively. The artificial intron is located between the downstream of exon 7 and the upstream of the combined exons 5–6‐7. The loxP sequence is written in red colour and the adenosine enclosed by a navy‐blue box represents an RNA splicing branch site. [file ADB-30-e70093-s002.pdf]

Supplementary figure 1

(intron4)
TCTTCTGGTG CTGCTATAGC CTGGTGGGCC TGATGACACG TTGCCCATCT GAGTTTCTCT ATTCCCCTAG
(exon5)
ACCAGAATGA GTGTATCATT GCCAACCCTG CCTTCGTGGT CTACTCCTCC ATCGTCTCGT TCTACGTGCC
CTTCATCGTC ACCCTGCTGG TCTATATCAA AATCTACATC GTTCTCCGCA AGCGTCGGAA GCGGGTCAAC
(intron5)
ACCAAGCGTA GCAGCCGAGC TTTCAGAGCC AACCTGAAGA CACCACTCAA G GTCTCCAAC CTCAGCCCCA
ACATGGGTCT CTACCTAAGC ATTATCAAGAG AGCTAATATT ACACATGGGT C GGGCCTGGA ACATTACAGT
AAGGTGGAAG TCTAGGCTGG AATAGTTTCC TGGGGTGAGA TGAGGGTGTA GACCTCAGTG GAGTGACAGA
ATTTTTACAA TGGGACTATG GTCCCTGGTG GGAGCTAAGT AGGATTCGCC ATGTGAAGAT CTTTGTGGCT
GTATGAAGCA ATTCTTACTT GATCTGATCC TTAGTTTCTC TATTCTTTCT CAGGTAGTTC TGCCTTTCTC
ATTATGGCCT CCATGTTTCC TTCCCCCTTC TTCCATCTTC TCTATACCAT TCTTTGTACC TGCTATGCTC
AGCTCTACCC TCCATGTACA CCCCTGCCTC CTCTTCCCCC ACTTTTTCCT CTGCTTTTTT TTTTTTTATC
ATAAAATCTC TCTCTTTCAC TCAACATTCT ACAATGAAGG CAGTAAGTGG GTATGGAAAT ATCAAGAACT
TATGGGTTAG ATACATCAGG TTCAAATCCT GGTCCTTTCA CCTTTTTAAA ATTGTGTGTC CTTAGGCATG
TTGGTTAGCT TCTATGAGCC TTGGTTTCCT CTTCTGTTAA GTGGGAGTCA TGTGCAATTC ACAGGGTTTT
CCTGAAGATT TGATGTGAGA AAGGATGTGA GCATGTGCCT AGCATGATCT TGAGTAGGTC CTCCACAAAG
AACAAGCTCC TTTTGCCTCT CACCTTGCCT GCCTCTTCTC ATCAGTGTAC CAGCCTTCCC TCTTGCCCGA
CTGTCTTCTG TGGCCCACAC TCCTCTAGTT GCTGAGCCCC TGACGAGAGT CTGAGTTCTC CATGGACTCC
TTTGTCTTGG GTTTCTATTC CCTTACCACT TCTGCTCTCC ACTGTCTTGT GTGACCCATG TGCTGGCTCA
(exon6)
CTCCACAG **GG CAACTGTACC CACCCTGAGG ACATGAAACT CTGCACCGTT ATCATGAAGT CTAATGGGAG**
(intron6)
TTTCCCAGTG AACAGGCGGA GAATG GTAAG TGTTCAGGTC AGGGACACAA TGCCATCTTT CTGCCTACCC
CCACCAATGG AGCCCACCAT CCCTGATCCT AGAATCTCAG CTATTTTAAG GGAGAAGTGT CTCCTGGCAC
CCCTACTTTG CTTCAGCTGA GAGCACATTT ACATTGTACA CTTCTGAGTA GAGAGAGATG CCTTAATTCT
GCAGGGCACA GATATGTTGA AGCTTGTTGG GGAAATAATC TACTCATAAA ACCTAGCAAA AGTGACTATA
CAGGCTAGTT CCACTCCTCA AACACCTCCA TGCTTCCTGA TATCACATCA AATCCTCAGG TAACCCTGGG
GAGTAATTCT TACTGGTCTT CAGCTCACAG CAGAGGTAGC CAAGGCTCAG AGTACAGGTA GCTCACCCAA
GTCTAAACAG TTAGTGAAAG AGTGCATATT GGAGCCAAGA TCTGTGTGAC TTCGAAATCC ACATCCAAGT
TTTTCCATTC AGCATGCACC ACCTTCTAGA TAGGCAGCTT CTGAGGCTGG GAGCTGGTGG CCAGCTGTAG
GGTGGGGACA GAGCAGAGCA GCCGGGGTAG ACAAGAAGCA TATTGTGAAC ATAGAACCTA TAAATGCAGA
CTTATAGCAT ACTTGAGCTC TCCATGGTGC TAGTTACAAC CAGAGTTCAA GAAGCAGGCA TTTCATCCCC
CAGCTAAGTT GGGATACATA AAATCCAGCA ATATGTCTCT TAGATAGGCA AGGTCCCTAC AATTGGCTGG
ATTCCAAGGA CAGGACTCTA ATGAACTATG TGTAATATAG GTACTAAAAC AGATCTCAAG TTATATTCAA
GGCAGCAGCC CATTTTTCAC CATCTGGGCA TATAGACTGT ATTTCAGAGG CTGATCCTAG ATCACAAAAG
GTCTGGGACA GTGGAGTCTA AAGAGGAACT GGGAAGCTAT GTGTGGATCC ATGTTGGAGC TTCTGGGTCA
GCCTACCAAG GAAGACCATA TGGCACTTCC AGATCAGCTC TATGCTTCGG AACCAAGCTA GTCAGCCCAC
ATATCCTTGG TGGTGCAGGT GCCTGTACTC CAGCCTGGCA GGGAGGATGG AGAACATGAT TTTTATGTAG
TTAGAATTAA GTTCTTGAAG GTGAAGCAAG ACTGAACTTC AGTCAGGGTT TATCTTAGCA GCTGAATTGG
TGTCCGGCCC TTAGCACTCA GGAAGTGTCA CTCAGGTTAT TTAAAGAGAA GTGGATGGGT GCTAGCATCC
AAGCATTTCT AGGGTATTGA GGCTTCACAG ATTGCTCTGG GCAGTGGTCC AGGAGTAACT GCCGTGAGTT
TGAGTGCTGT ATTCAGATGG TCCCTGCCTC CCAGTGATGG CTCTCTTTGT GTTCCTGGCA GAGAAGCCAT
GCTGTTTGCA AGAGTGTGCA GAGGGGCAGG AGGACCGAGG AGGCTTGGTG GCTTCTGCCC AGGAATTTCA
(exon7)
TCCTAAGTTC TCTCTCTCTG CTTCAG **GATG CTGCCCGCCG AGCTCAGGAG CTGGAAATGG AGATGCTGTC**
AAGCACCAGC CCCCCAGAGA GGACCCGGTA TAGCCCCATC CCTCCCAGTC ACCACCAGCT CACTCTCCCC
GATCCATCCC ACCACGGTCT ACATAGCAAC CCTGACAGTC CTGCCAAACC AGAAAAGAAT GGGCATGCCA
AGATTGTCAA TCCCAGGATT GCCAAGTTCT TTGAGATCCA GACCATGCCC AATGGCAAAA CCCGGACCTC
CCTTAAGACG ATGAGCCGCA GGAAGCTCTC CCAGCAGAAG GAGAAGAAAG CCACTCAGAT GCTTGCCATT
(artificial intron including loxP)
GTTCTTG GTAC GTCGTATAAC TTCGTATAAT GTATGCTATA CGAAGTTATG TTG A TGGTTC TTCCATATTC
(combined exons 5-6-7) (exon5)
CCCTAG **ACCA GAATGAGTGT ATCATTGCCA ACCCTGCCTT CGTGGTCTAC TCCTCCATCG TCTCGTTCTA**
CGTGCCCTTC ATCGTCACCC TGCTGGTCTA TATCAAAATC TACATCGTTC TCCGCAAGCG TCGGAAGCGG
(exon6)
GTCAACACCA AGCGTAGCAG CCGAGCTTTC AGAGCCAACC TGAAGACACC ACTCAAG GGC AACTGTACCC
ACCCTGAGGA CATGAAACTC TGCACCGTTA TCATGAAGTC TAATGGGAGT TTCCCAGTGA ACAGGCGGAG
(exon7)
AATG GATGCT GCCCGCCGAG CTCAGGAGCT GGAAATGGAG ATGCTGTCAA GCACCAGCCC CCCAGAGAGG
ACCCGGTATA GCCCCATCCC TCCCAGTCAC CACCAGCTCA CTCTCCCCGA TCCATCCCAC CACGGTCTAC
ATAGCAACCC TGACAGTCCT GCCAAACCAG AAAAGAATGG GCATGCCAAG ATTGTCAATC CCAGGATTGC
CAAGTTCTTT GAGATCCAGA CCATGCCCAA TGGCAAAACC CGGACCTCCC TTAAGACGAT GAGCCGCAGG
(intron downstream of the combined exons 5-6-7)
AAGCTCTCCC AGCAGAAGGA GAAGAAAGCC ACTCAGATGC TTGCCATTGT TCTTG GTGAG TAAGCTCTGG
GTGATGGCCA CAGCCCTGCC GTGCCCAGCC CTGGCAACAT CCCAGCAGGT CCTAGACTCT GCCTTGTTG

Legend for Supplementary figure 1

The sequence of the artificial intron, the combined exons 5-6-7 and the surrounding regions included in the targeting vector. The sequences from 70 bp at the 3' end of intron 4 of the mouse Drd2 (D2R) gene to 85 bp of the 3’ downstream of the combined exons 5-6-7 used in the targeting vector are described. The sequences of exons 5, 6 and 7 are written in blue, orange and green color, respectively. The artificial intron is located between the downstream of exon 7 and the upstream of the combined exons 5-6-7. The loxP sequence is written in red color and the adenosine enclosed by a navy-blue box represents an RNA splicing branch site.
