# Supplementary material for: Dopamine D2S/D2L Receptor Regulation of Alcohol‐Induced Reward and Signalling
Source: Addict Biol. 2025 Nov 15;30(11):e70093. doi: 10.1111/adb.70093 (PMC12619067; doi:10.1111/adb.70093)
Supplement: Supplementary file 2 — Table S1: List of primary and secondary antibodies used in the study. SFig. 4. EtOH‐induced CPP was assessed in three genotypes of mice. These are the same data used for figure 4 in the main text except that the statistical analysis was done using Student's t‐test (unpaired). CPP was significantly increased in EtOH‐treated D2L KO mice (D2LKO EtOH) as compared to saline‐treated D2L KO mice (D2LKO Saline) (**p = 0.001; t‐test, unpaired). CPP was also significantly increased in EtOH‐treated WT mice (WT EtOH) as compared to saline‐treated WT (WT Saline) (*p = 0.019; t‐test, unpaired). There was no significant change in CPP between EtOH‐treated D2S KO mice (D2SKO EtOH) and saline‐treated D2S KO mice (D2SKO Saline) (p = 0.201; t‐test, unpaired; ns = not significant). SFig. 7. Relative protein expression levels of Akt in the striatum of WT, D2S KO, and D2L KO mice treated with EtOH or saline were analysed by Western blotting. No significant differences in Akt protein levels were observed between any genotype pairs. [WT Saline vs. WT EtOH, p = 0.7593; D2SKO Saline vs. D2SKO EtOH, p = 0.9900; D2LKO Saline vs. D2LKO EtOH, p = 0.6074; overall, F(2, 18) = 0.5788, p = 0.5707, two‐way ANOVA with post hoc Šidák's test]. [file ADB-30-e70093-s001.pdf]

Supplementary Materials

Supplementary Table 1: List of primary and secondary antibodies used in the study

Primary Antibodies			
Protein	Antibody	Company Name, Country	Dilution
D1R	Mouse anti D1DR	Santa Cruz Bio., USA	1:1000
D2R	Rabbit anti D2DR	Proteintech, USA	1:1000
Phospho-Akt (Ser473)	Rabbit anti Phospho-Akt (Ser473)	Cell Signaling, USA	1:1000
Akt	Rabbit anti Akt	Cell Signaling, USA	1:1000
GAPDH	Mouse anti GAPDH	Santa Cruz Bio., USA	1:1000
CB1R	Rabbit anti CB1R	Proteintech, USA	1:1000
Secondary Antibodies			
Antibody		Company Name	Dilution
HRP conjugated anti-rabbit IgG		Invitrogen, USA	1:10000
HRP conjugated anti-mouse IgG		Invitrogen, USA	1:10000

SFig. 4 (Supplementary figure for Fig. 4)

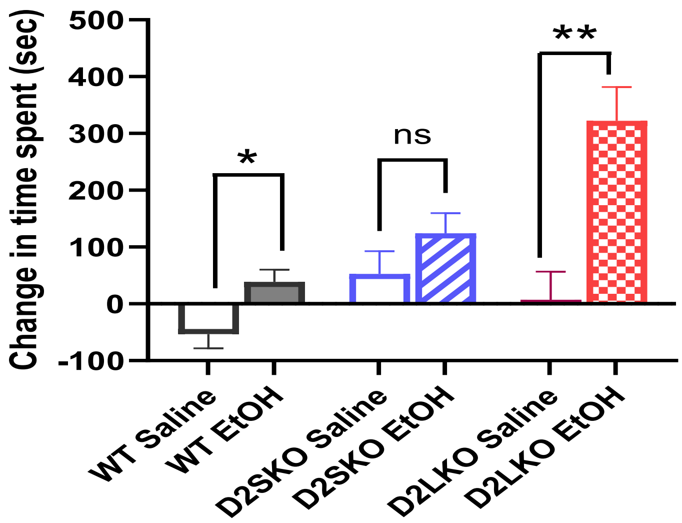

SFig. 4. EtOH-induced CPP was assessed in three genotypes of mice. These are the same data used for figure 4 in the main text except that the statistical analysis was done using Student's *t*-test (unpaired). CPP was significantly increased in EtOH-treated D2L KO mice (D2LKO EtOH) as compared to saline-treated D2L KO mice (D2LKO Saline) (***P* = 0.001; *t*-test, unpaired). CPP was also significantly increased in EtOH-treated WT mice (WT EtOH) as compared to saline-treated WT (WT Saline) (**P* = 0.019; *t*-test, unpaired). There was no significant change in CPP between EtOH-treated D2S KO mice (D2SKO EtOH) and saline-treated D2S KO mice (D2SKO Saline) (*P* = 0.201; *t*-test, unpaired; ns = not significant).

SFig. 7 (Supplementary figure for Fig. 7)

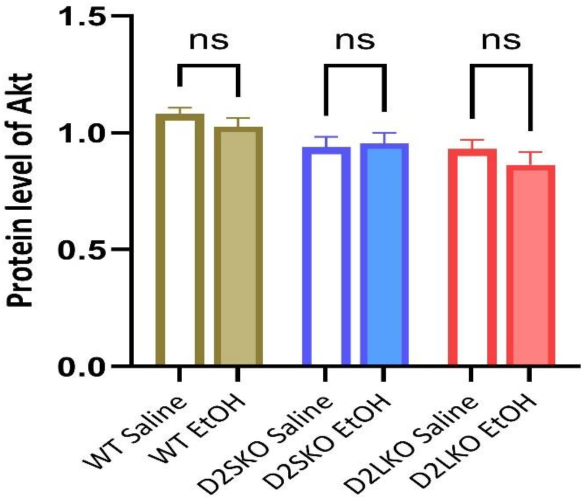

SFig. 7. Relative protein expression levels of Akt in the striatum of WT, D2S KO, and D2L KO mice treated with EtOH or saline were analyzed by Western blotting. No significant differences in Akt protein levels were observed between any genotype pairs. [WT Saline vs. WT EtOH, P = 0.7593; D2SKO Saline vs. D2SKO EtOH, P = 0.9900; D2LKO Saline vs. D2LKO EtOH, P = 0.6074; overall, $F(2,18) = 0.5788$, P = 0.5707, two-way ANOVA with post-hoc Šidák's test]
